# Biomechanical and neuromuscular differences between professional and varsity football players during countermovement and approach jumps

**DOI:** 10.1371/journal.pone.0336672

**Published:** 2025-12-01

**Authors:** Gabriela Garcia, Rafaela Bucheli, Jose Tomas Castillo, Julian Fernandez, Alejandra de la Torre, Andres Leon Fierro, Wendy Montiel, Paul G. Arauz

**Affiliations:** 1 Departamento de Ingeniería Industrial, Colegio de Ciencias e Ingenierías, Universidad San Francisco de Quito USFQ, Quito, Ecuador; 2 Instituto de Ciencias del Ejercicio, Universidad San Francisco de Quito USFQ, Quito, Ecuador; 3 Escuela de Medicina, Colegio de Ciencias de la Salud, Universidad San Francisco de Quito USFQ, Quito, Ecuador; 4 Club Deportivo Independiente del Valle, Quito, Ecuador; 5 Department of Orthopaedics, Renaissance School of Medicine, Stony Brook University, New York, New York, United States of America; ASPIRE Academy for Sports Excellence, QATAR

## Abstract

This study aimed to compare lower-body kinematics, muscle activity, performance, and vertical ground reaction force (GRFz) metrics between professional and varsity Ecuadorian football players during countermovement jumps with arm swing (CMJ_AS_) and approach jumps (AJ). These two jump types were selected because they closely reflect common football-specific movement patterns; CMJ_AS_ simulates vertical jumps with arm drive, while AJ mimics explosive jumping after a run-up. Forty athletes (20 professional, 20 varsity; equally distributed by sex) were assessed using synchronized motion capture, force plates, and surface electromyography. Professional males demonstrated greater force production efficiency, defined here as the ability to generate higher concentric average GRFz, higher braking peak GRFz, shorter movement durations, and higher rates of force development in the unloading, yielding and braking phases, while achieving similar jump heights and modified reactive strength index (RSI mod) to varsity players during CMJ_AS_. However, they also experienced higher impact forces in the AJ. Among females, professionals outperformed varsity players in both jumps, achieving greater jump heights, higher RSI mod scores, and increased concentric average GRFz. They also exhibited higher landing impact forces, loading rates, and asymmetric vastus medialis mean activation during landing; patterns commonly associated with elevated injury risk. Across groups, joint range of motion (ROM) and muscle activation patterns varied by phase, with professionals generally showing more proximal muscle activation and neuromuscular control. Asymmetries in ROM and muscle activation were more pronounced among professional females, particularly during AJ, suggesting task-specific adaptations that may also influence injury susceptibility. These findings underscore the importance of a comprehensive biomechanical assessment to inform injury screening and targeted strategies for injury risk reduction in competitive football.

## Introduction

Football, or soccer, is one of the most widely played sports worldwide, with over 130,000 professional players and more than 4,400 professional clubs (FIFA, 2024). Player performance in football involves both on-ball actions (e.g., passing, shooting) and off-ball movements (e.g., sprinting, positioning), both of which are crucial for overall success in the game [1]. High-intensity movements such as sprints play a key role in goal-scoring opportunities [2], while other actions, like heading, occur frequently at the elite level [3]. The effectiveness of these movements is influenced by physiological and biomechanical factors, including muscle activation, joint ROM, and lower-body strength, which collectively determine an athlete’s physical capabilities [4].

Muscle activation and joint range of motion (ROM) are key biomechanical variables that influence both athletic performance and injury risk in football. Their relevance stems from the demands of sport-specific movements that require coordinated force production, joint mobility, and neuromuscular control. For instance, the vastus medialis and rectus femoris are primarily engaged during movements requiring upper-leg force production, such as sprinting, jumping for headers, and executing powerful shots on goal [5–8]. Meanwhile, the biceps femoris and medial gastrocnemius contribute to lower-leg stability during actions like landing from a jump, decelerating during a sprint, and maintaining balance while changing direction or pivoting during defensive maneuvers [9–11]. In addition, kicking mechanics are highly asymmetric, with significant differences in muscle activation between the supporting and kicking limbs [12]. While some asymmetries reflect functional adaptations, others may elevate injury risk [13,14]. Similarly, limited flexibility and restricted ROM in the lower limbs have been associated with reduced kicking speed and compromised performance [15,16]. Taken together, muscle activation and ROM offer important insights into physical readiness, interlimb coordination, and potential risk factors for lower-limb injury [17–19].

Given the importance of muscle activation and ROM in football performance and the association with injury risk, it is essential to use reliable tools to evaluate these characteristics. Biomechanical assessments serve as such tools by offering objective measures of neuromuscular function, joint mobility, and lower-body mechanics [20,21]. These assessments are widely used to evaluate muscle activation [22], ROM [23,24], vertical ground reaction force (GRFz) variables [25,26], and traditional performance metrics (including jump height, jump momentum, and the reactive strength index modified (RSI mod)) [27] in football players, as they provide critical insights into performance capabilities and injury risks [27]. Understanding the biomechanics of the whole body and individual joints at the time of injury is a key step toward developing effective strategies that contribute to injury risk reduction [28]. By identifying altered or risky movement patterns, such as excessive knee valgus during landing, asymmetrical limb loading, or prolonged ground contact during cutting, these assessments can help tailor training programs to reduce the incidence of common injuries, such as those to the knee and ankle. In elite football, nearly 90% of anterior cruciate ligament (ACL) injuries occur without direct knee contact, often during movements like landing, cutting, or pressing, highlighting the importance of screening for biomechanical deficits that increase injury susceptibility [29]. Muscular imbalances, for example, have been associated with an increased likelihood of hamstring strains [30,31] and ACL injuries [32], while asymmetries in ROM have been linked to hip and lower back pain [33,34]. Performance testing also plays a crucial role in player monitoring, allowing practitioners to compare athletes’ ROM against established reference values, such as those from professional football players [35]. This comparative approach is widely used across different sports to assess training progress and injury risk [36].

Jumping performance is particularly relevant in football, as it is strongly correlated with agility [37], sprint speed [38,39], and change-of-direction ability [40]. Jumping is not only one of the most popular training modalities among football players [41], but also a widely used method for assessing lower-body function in athletes [42–44]. In this study, we selected the countermovement jump with an arm swing (CMJ_AS_) and the approach (run-up) jump (AJ) because they closely replicate common football-specific actions, such as leaping for a header after a run-up or jumping from a stationary stance with full-body coordination. These tasks involve both vertical propulsion and coordinated arm-leg movement making them ecologically valid tests of functional performance in football. Vertical jump tests like the CMJ_AS_ and AJ are used to assess explosive strength, defined here as the capacity to generate force rapidly (i.e., neuromuscular power), which is often inferred from variables such as peak force, rate of force development (RFD), and jump height. Both jumps have demonstrated high test-retest reliability, with intraclass correlation coefficients (ICCs) typically ranging from 0.85 to 0.96 for jump height and force metrics in athletic populations [45–47]. These tests provide critical information regarding an athlete’s explosive strength and neuromuscular control, making them essential tools in football performance analysis and identifying potential injury risk factors [48–50].

While traditional CMJ protocols often restrict arm movement to isolate lower-limb performance and improve experimental control, recent findings suggest that including an arm swing offers a more holistic assessment of athletic performance. CMJs performed with an arm swing have been shown to produce greater jump height and propulsive power, reflecting enhanced neuromuscular coordination and more effective use of the stretch-shortening cycle [51]. In football, movements like jumping to head a ball or competing in aerial duels inherently involve coordinated arm actions. Therefore, including the CMJ_AS_ in the present study was a deliberate choice to enhance ecological validity. From a biomechanical perspective, the use of arm swing introduces additional degrees of freedom, promoting a more effective proximal-to-distal extension strategy and increasing vertical impulse at the hip and ankle joints [52]. By evaluating the CMJ_AS_ rather than CMJ without arm swing, we aimed to capture full-body performance characteristics and neuromuscular control strategies that are more representative of real-game demands in football. Additionally, although CMJs without arm swing emphasize a force-driven strategy, the arm swing condition (CMJ_AS_) relies more on time-driven mechanisms, such as increased countermovement depth and duration, leading to enhanced propulsive impulse and jump height, without compromising measurement reliability [53]. Thus, the inclusion of arm swing in jumping assessments may provide a more comprehensive representation of the complex, coordinated, and dynamic demands inherent to football performance.

Despite the growing body of research on football biomechanics, there is limited data on elite Ecuadorian players. While some studies have been conducted in other Latin American countries, such as Colombia [54], most research focuses on European population and sex-based differences in muscle activation and ROM [55,56]. In addition, few studies have directly compared professional and varsity football players, particularly in relation to biomechanical risk factors linked to injury. Understanding these differences is essential not only for optimizing training programs, but also for improving injury reduction strategies and tailoring screening protocols. This is particularly relevant when considering geographical factors such as altitude, climate, and access to sports science infrastructure, all of which may influence physical performance and injury profiles in football athletes from different regions [57,58]. In addition, population-level characteristics, such as ethnicity-related variations in anthropometry and neuromuscular control, may also influence biomechanical performance and injury risk [59,60]. These factors underscore the importance of region-specific data to inform training and injury reduction strategies.

Thus, the primary aim of this study is to compare the biomechanical and neuromuscular characteristics of professional and varsity male and female football players during two common vertical jump tasks: the CMJ_AS_ and the AJ. These tasks simulate sport-specific movements relevant to injury risk, such as landing from a jump or executing rapid directional changes. Landing, in particular, is a known mechanism for non-contact ACL injuries, especially in male football players, where valgus knee loading and inadequate neuromuscular control are commonly observed [29]. To achieve these objectives, we employed synchronized motion capture, force plate analysis, and surface electromyography to evaluate joint kinematics, ground reaction forces, and muscle activation patterns during each jump phase. These integrated assessments enable the identification of neuromuscular control strategies, interlimb asymmetries, and loading characteristics that may signal elevated injury susceptibility. The findings can inform sex- and level-specific screening, training, and injury reduction strategies. Moreover, this research will contribute to a broader understanding of the regional and competitive-level differences in football biomechanics, laying the groundwork for future studies in underrepresented populations. Given the evidence that biomechanical characteristics such as ROM, force production, and neuromuscular activation vary across sport levels and sexes [25,61–64], we sought to test whether such differences are observable in Ecuadorian football players. Using validated biomechanical tests, this study allows for the detection of potentially modifiable risk factors and performance indicators between professional and varsity groups. Hence, this study seeks to test the following null hypotheses:

- There are no significant differences in lower-body joint ROM between professional and varsity football players during the CMJ_AS_ and AJ.
- There are no significant differences in mean or peak muscle activation levels between professional and varsity football players during the CMJ_AS_ and AJ.
- There are no significant differences in performance or vertical ground reaction force metrics between professional and varsity football players during the CMJ_AS_ and AJ.

## Materials and methods

### Participants

Forty football players (20 professional and 20 college varsity) participated in this study, with an equal distribution of sex in each category. This sample provided an achieved statistical power of 92%, calculated post hoc using G*Power 3.1.9.7 with an alpha level of 0.05 and a partial eta-squared (ηp2) effect size of 0.08, which represents the average of the effect sizes observed across key outcome variables in this study. Professional players were recruited from Club Independiente del Valle, an elite professional football club in Ecuador, while varsity players were recruited from Universidad San Francisco de Quito football team. Notably, in Ecuador, male varsity football teams are considered semiprofessional due to the high demand and competitive nature of the sport. This may differ from the typical collegiate classification in North America and Europe, where varsity athletes may train and compete under different conditions. We acknowledge this distinction and encourage readers to consider it when comparing our findings with studies conducted in other countries.

All participants reported being free from recent musculoskeletal injuries or pain and were actively engaged in their regular training schedules. Before participation, all players provided written informed consent, as approved by the Ethics Committee of the Universidad San Francisco de Quito (protocol #2023−088IN). This study adhered to the ethical principles outlined in the Declaration of Helsinki. Participants were recruited from January 30, 2024 until May 30, 2024. Participants’ demographic characteristics are presented in Table 1.

**Table 1:** Participant demographic data.

	Professional Males		Varsity Males		Professional Females		Varsity Females	
	Mean	SD	Mean	SD	Mean	SD	Mean	SD
**Mass (kg)**	75.33	5.50	66.80	10.28	59.01	6.65	59.86	6.32
**Height (cm)**	179.67	4.15	177.82	8.27	163.94	8.09	160.73	4.38
**Age (years)**	21.30	4.45	20.36	1.36	23.40	4.27	18.80	1.62

### Procedure

Participants attended a single two-hour experimental session at the laboratory during the in-season period. All participants reported to the laboratory at least two hours after completing their routine morning field training session and did not engage in any resistance or strength training prior to testing. Upon arrival, they were briefed on the study protocol, provided informed consent, and completed demographic data collection. Surface electromyography (EMG) electrodes were placed on selected lower-limb muscles following standard preparation protocols. A 10-minute warm-up session was conducted, consisting of approximately 5 minutes of dynamic mobility drills (including walking lunges, leg swings, hip circles, and bodyweight squats), followed by 5 minutes of treadmill running at a self-selected moderate pace, based on each athlete’s perception of moderate effort.

Subsequently, submaximal voluntary isometric contractions (subMVCs) were performed to obtain reference values for EMG data normalization, as commonly performed in EMG studies [65–68]. Each participant completed three 10-second repetitions per exercise, with one-minute rest intervals between repetitions and a three-minute rest period between different exercises. The loads used to elicit submaximal contractions were fixed (e.g., 40 kg for resistance band exercises, 30 kg for the weighted bar) and selected by the teams’ sports medicine staff based on their knowledge of each team’s strength levels, ensuring the effort remained safely submaximal for all players. The exercises included prone leg curls against 40 kg resistance bands for the biceps femoris, bilateral seated leg raises (knee extension) against 40 kg resistance bands for the vastus medialis and rectus femoris, and bilateral standing plantar flexion while holding a 30 kg weighted bar in front of the body with extended arms, in a position similar to the starting posture of a deadlift, for the gastrocnemius medialis [69]. Although each limb was connected to an individual 40 kg resistance band, all isometric exercises were performed bilaterally, with both legs contracting simultaneously. EMG values obtained during these subMVCs served exclusively for normalization purposes, allowing all activation data during the jump tasks to be expressed as a percentage of this reference activity. Following these exercises, reflective markers were placed on relevant lower-body landmarks for motion capture.

Participants were then instructed on two movement tasks: the CMJ_AS_ and AJ (initiated with the dominant leg). To encourage maximal jump height, a ball was suspended above participants, simulating a heading scenario as suggested in previous research [70]. Participants were explicitly instructed to “jump as high as you can and try to touch the ball with your head” during each trial. This cue was intended to direct their attention toward vertical displacement while maintaining ecological validity. Each participant performed at least three familiarization trials before completing three recorded experimental trials for each task. The order of jump conditions was randomly assigned, and a two-minute rest interval was provided between trials. Throughout testing, synchronized EMG, motion capture, and force plate systems were used to collect biomechanical and neuromuscular data.

### Apparatus and measures

#### EMG.

Muscle activity was recorded bilaterally of the biceps femoris, rectus femoris, vastus medialis, and gastrocnemius medialis using wireless bipolar surface EMG sensors (Delsys Inc, Boston, MA). Trigno EMG sensors sampled the gastrocnemius medialis at 1926 Hz, while Avanti EMGs captured the biceps femoris, rectus femoris, and vastus medialis at a rate of 2148 Hz. Prior to sensor attachment, the skin over the muscle sites was prepared by shaving and treating it with an abrasive gel (Skin Prep Gel, Nuprep®, Aurora, USA) to minimize impedance and optimize electrode contact. Sensor placement adhered to the SENIAM (Surface ElectroMyoGraphy for the Non-Invasive Assessment of Muscles) guidelines for standardized positioning.

EMG data were processed in MATLAB (MathWorks, Inc., Natick, MA). Raw signals were first detrended to remove baseline offset that could bias amplitude estimation. A fourth-order Butterworth bandpass filter (30–300 Hz) was then applied to minimize motion artifacts typically found below 30 Hz and reduce high-frequency noise above 300 Hz, as recommended for dynamic sports movements [71,72]. Signal quality was verified using Fast Fourier Transform analysis. Root mean square (RMS) values were computed using a 250-ms moving window with 50% overlap, which offers an optimal balance between smoothing and temporal resolution for dynamic tasks like jumping [71,73,74]. EMG amplitudes were then normalized to submaximal voluntary contraction values obtained during isometric exercises, as described in the procedure section. Finally, mean and peak muscle activation were calculated for each phase of the jumping tasks.

#### Motion capture.

**Fig 1:**
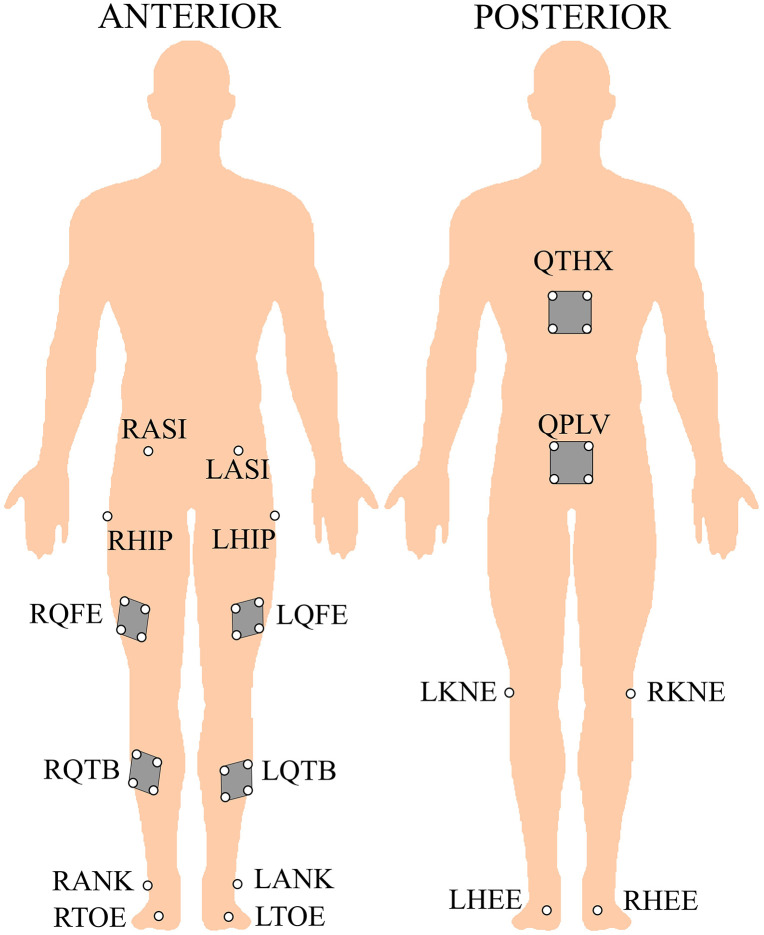
Lower-body markers set.

Lower-body kinematic data were captured using a 10-camera motion capture system (Vicon MX, Oxford, UK) operating at a sampling frequency of 100 Hz. Reflective spherical markers (10 mm in diameter) were placed on participants at key anatomical landmarks. Six clusters, each comprising four markers, and an additional 12 individual markers were positioned to define and track the thorax-pelvis, hip, knee, and ankle movements, as illustrated in Fig 1. Three-dimensional joint angles were calculated relative to a relaxed standing posture, designated as the neutral zero reference position [75–78]. Segmental and joint motions were determined using a Cardan YXZ rotation sequence, with the right-hand rule applied to define rotation direction [75,76,79]. The kinematic data were exported and processed in MATLAB (MathWorks, Inc., Natick, MA) using a custom-built program tailored for this study, where ROM angles were calculated separately for each phase of the jumping tasks.

#### Force plates.

Ground reaction forces (GRFs) were recorded using two force plates (Kistler, Winterthur, Switzerland) integrated with Noraxon MR3 software (Noraxon, USA) at a sampling frequency of 1500 Hz. These force data were used to segment the movements into distinct phases based on previous guidelines [80] using a custom MATLAB program.

**Fig 2:**
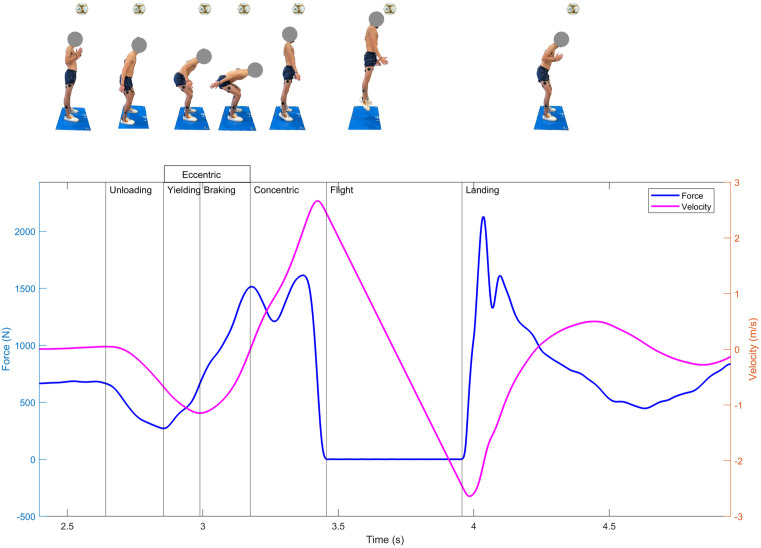
Phases of the countermovement jump with arm swing (CMJ_AS_) and corresponding force-velocity curves from a representative subject.

For the CMJ_AS_, the phases were defined as unloading (P1), eccentric [yielding and braking] (P2), concentric (P3), flight (P4), and landing (P5), consistent with prior research [27,81,82], as shown in Fig 2. The unloading phase began when the center of mass (COM) started to descend. This point was estimated as the instant when the vertical ground reaction force (GRFz) deviated beyond the mean by ±5 times the standard deviation (SD) of the average force measured during the weighing phase, when the participant stood still for about three seconds [80]. Body weight was determined from one second of quiet standing within this period. During the unloading phase, the participant reduced force against the ground, effectively “unweighting” in preparation for the countermovement. This phase ended when the GRFz reached its lowest local magnitude (i.e., local minimum) (Fig 2).

The eccentric phase followed, characterized by the downward motion of the body as the hips, knees, and ankles flexed. During this phase, the muscles lengthened under tension, storing elastic energy for the subsequent concentric action. The eccentric phase was further divided into two subphases: the yielding phase, which ended at the instant of peak negative velocity, and the braking phase, which continued until the velocity of the COM reached 0 m/s [80]. The concentric phase began immediately after the eccentric phase and continued until takeoff, defined as the point when the GRFz fell below the 20 N threshold, indicating that the feet had left the ground. This phase involved upward movement, during which the hips, knees, and ankles extended to generate force and propel the participant off the ground. The flight phase commenced at takeoff and ended at landing, marked by the GRFz rising above the 20 N threshold upon ground contact. Finally, the landing phase began at the moment of ground contact and continued until the participant regained stability, defined as the GRFz returning to body weight.

Performance variables included jump height (m), jump momentum (kg·m/s), and the reactive strength index modified (RSI mod). Jump height was calculated as the square of the velocity at takeoff divided by twice the acceleration due to gravity. Jump time was defined as the ground contact duration from the start of movement to takeoff. RSI mod was calculated by dividing jump height by jump time [27].

Additionally, the duration of each jump phase (unloading, eccentric [yielding and braking], concentric, flight, and landing) was calculated in seconds. These vertical ground reaction force (GRFz) metrics were calculated following previously outlined equations [27,82]. Ground reaction force variables were normalized to body weight (BW) and expressed in either percentage or units of BW or BW per second, depending on the metric.

Unloading GRFz, was defined as the GRFz at the end of the unloading phase (Unloading End GRFz) and was expressed as a percentage of body weight (%BW). Unloading rate of force development (RFD) was calculated as the difference between the Unloading End GRFz and the initial GRFz during the unloading phase, divided by the duration of the phase, and then normalized to BW (BW/s). Yielding RFD was computed as the difference between the GRFz when the local peak negative velocity was achieved (Yielding GRFz) and the Unloading End GRFz, divided by the duration of the phase, and then normalized to BW (BW/s).

Braking GRFz, also referred to as the amortization force or the GRFz at zero velocity, was defined as the peak vertical ground reaction force at the transition between the eccentric and concentric phases, and was expressed in units of BW. Braking RFD was calculated as the difference between the GRFz at the end of the braking phase and the Yielding GRFz, divided by the duration of the phase, and normalized to BW (BW/s). Concentric average GRFz was calculated as the mean vertical ground reaction force during the concentric phase and normalized to BW, yielding values in relative units of BW (units of BW). Momentum at landing was calculated by multiplying body mass by vertical velocity at ground contact and was expressed in kg.m/s. Impact peak GRFz was defined as the maximum GRFz occurring during the loading phase of landing, which is the period from initial ground contact until the GRFz reached its peak [82], and was expressed in units of BW. Impact average GRFz was calculated as the mean of GRFz from the moment of ground contact to the point of peak GRFz, also expressed relative to BW (units of BW). Lastly, the loading rate was determined by subtracting the GRFz at ground contact from the peak GRFz, dividing by the duration of the loading phase, and normalizing the result to BW per second (BW/s).

**Fig 3:**
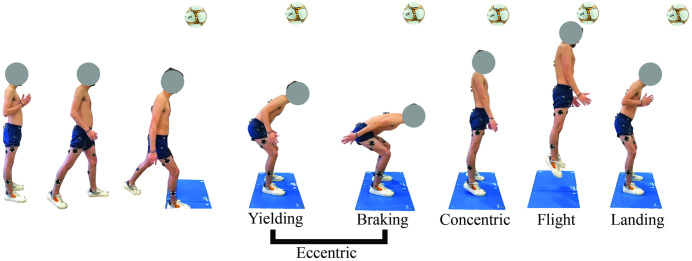
Phases of the approach jump (AJ).

In the AJ, the unloading phase was absent because the movement began with foot contact on the force plate. The remaining phases; eccentric (P2), concentric (P3), flight (P4), and landing (P5); were defined in the same manner as for the CMJ_AS_ (Fig 3). For this jump less metrics were selected to facilitate comparisons: jump height, braking peak GRFz, concentric average GRFz, concentric peak GRFz, impact peak GRFz and average impact GRFz. All metrics were expressed in units of BW.

### Data analysis

Statistical analyses were performed using mixed models with a variance-components covariance structure and residual maximum likelihood estimation in SAS Studio (SAS Institute Inc.). Participants were treated as random effects in all models. To assess bilateral symmetry, metrics from both the dominant and non-dominant legs were compared. For EMG and motion capture metrics, player category (Category: professional vs. varsity) and leg (Side: left vs. right) were included as fixed effects, and a 2x2 mixed model was used to test both main effects and interactions. For performance and GRFz metrics (measured at the body level), only Category was included as a fixed effect. All data met the assumption of normality, and statistical significance was set at α = 0.05. Post hoc comparisons were conducted using least square mean differences, with p-values adjusted using the Tukey-Kramer method to control for Type I error in multiple testing. Partial eta-squared pseudo-effect size (ηp^2^) were calculated using a method suitable for mixed models [83] and interpreted as small (ηp^2^ = .01), medium (ηp^2^ = .06), and large (ηp^2^ = .14). Effect size interpretation was only applied to results that met the significance threshold (p < 0.05); non-significant findings were not interpreted based on effect size.

## Results

### Joint range of motion: CMJ_AS_

Mixed models fixed effects results and descriptive statistics, including means and standard deviations for sagittal plane joint ROM data across the five phases of the CMJ_AS_, are presented in Tables 2 and 3. No significant Category × Side interactions were found in either male or female athletes. A significant main effect of player category was observed in males, with differences at the ankle, knee, and hip across multiple phases with small to medium effect sizes (Table 2), while no significant differences were detected at the thorax-pelvis angle.

**Table 2:** Descriptive statistics and fixed effects statistical results for sagittal plane joint range of motion (ROM) in the knee, hip, and ankle (degrees) for male and female professional and varsity football players during countermovement jump with arm swing (CMJ_AS_).

		Joint Angles Mean±SD%				Fixed Effects		
Males		Professional		Varsity		*p*-value (ηp^2^)		
Joint	Phase	Left	Right	Left	Right	Category	Side	Category x Side
Knee	P1	22.08 ± 6.7	21.42 ± 7.34	17.97 ± 6.23	17.11 ± 5.71	.05(.001)	.39(.0001)	.91(<.0001)
	P2	53.1 ± 12.37	54.44 ± 13.2	65.52 ± 12.85	65.52 ± 13.3	.01(.05)*	.68(.001)	.68(.001)
	P3	76.2 ± 10.39	76.29 ± 12	84.86 ± 9.52	83.91 ± 11.09	.04(.03)*	.74(.001)	.68(.001)
	P4	16.47 ± 6.81	13.33 ± 5.78	11.7 ± 5.65	10.58 ± 4.45	.03(.04)*	.01(.05)*	.24(.01)
	P5	68.12 ± 19.21	68.58 ± 18.3	63.02 ± 11.47	60.81 ± 10.6	.33(.01)	.52(.003)	.32(.01)
Hip	P1	24.64 ± 6.47	24.77 ± 6.87	16.25 ± 5.88	16.11 ± 5.98	<.0001(.01)*	.99(<.0001)	.88(<.0001)
	P2	46.73 ± 12.67	47.03 ± 12.54	60.94 ± 13.62	61.33 ± 13.78	.004(.06)*	.82(.0004)	.97(<.0001)
	P3	60.31 ± 8	61.16 ± 8.83	70.6 ± 5.9	71.26 ± 6.89	.001(.1)*	.38(.006)	.91(<.0001)
	P4	10.42 ± 4.04	10.4 ± 3.14	11.58 ± 4.52	11.18 ± 4.1	.44(.005)	.71(.001)	.74(<.0001)
	P5	48.9 ± 23.87	50.29 ± 23.29	40.96 ± 16.94	40.28 ± 16.62	.31(.01)	.81(.0004)	.47(.004)
Ankle	P1	7.29 ± 3.39	7.51 ± 3.33	7.12 ± 4.22	6.13 ± 3.21	.47(.002)	.47(.0002)	.25(<.0001)
	P2	15.96 ± 5.02	15.79 ± 4.43	18.73 ± 5.58	19.64 ± 5.82	.10(.02)	.52(.003)	.35(.007)
	P3	51.72 ± 8.03	50.78 ± 8.03	57.48 ± 8.65	56.81 ± 9.79	.03(.03)*	.51(.003)	.91(.0001)
	P4	17.34 ± 8.89	18.14 ± 8.99	13.53 ± 6.92	11.75 ± 8.22	.07(.02)	.72(.001)	.30(.009)
	P5	47.9 ± 9.22	46.57 ± 9.88	56.34 ± 8.77	53.91 ± 10.79	.02(.04)*	.11(.02)	.59(.002)
**Females**		**Professional**		**Varsity**		***p*-value (ηp**^**2**^)		
**Joint**	Phase	**Left**	**Right**	**Left**	**Right**	**Category**	**Side**	**Category x Side**
Knee	P1	18.2 ± 7.93	18.33 ± 7.77	21.75 ± 7.75	21.84 ± 9.02	.15(.01)	.95(<.0001)	.86(<.0001)
	P2	48.75 ± 11.61	50.16 ± 10.82	45.78 ± 14.01	47.39 ± 15.27	.55(.002)	.32(.01)	.94(<.0001)
	P3	74.2 ± 12.47	75.49 ± 11.65	73.82 ± 7.35	72.8 ± 8.99	.67(.001)	.92(<.0001)	.38(.01)
	P4	18.2 ± 5.36	16.92 ± 4.95	16.86 ± 5.98	17.73 ± 5.74	.87(.0002)	.82(.0004)	0.24(.01)
	P5	68.5 ± 15.6	70.42 ± 16.12	66.63 ± 12.48	65.35 ± 11.74	.53(.003)	0.83(<.0001)	.28(.01)
Hip	P1	21.94 ± 8.69	22.44 ± 8.42	24.87 ± 9.54	25.1 ± 9.49	.26(.01)	.74(.001)	.91(.0001)
	P2	50.36 ± 11.83	50.77 ± 12	47.75 ± 16.73	48.77 ± 17.77	.65(.001)	.72(.001)	.88(.0002)
	P3	63.7 ± 9.55	64.35 ± 10.04	65.1 ± 7.01	65.08 ± 6.6	.75(<.0001)	.71(.001)	.68(.001)
	P4	12.99 ± 4.96	13 ± 4.95	10.49 ± 5.65	10.86 ± 5.14	.22(.01)	.78(.001)	.79(<.0001)
	P5	64.86 ± 21.86	65.71 ± 22.07	48.86 ± 19.09	48.41 ± 16.7	.05(.03)	.88(.0001)	.65(.001)
Ankle	P1	5.92 ± 3.44	6.21 ± 3.28	9.38 ± 4.36	8.37 ± 3.95	.02(.04)*	.63(.001)	.31(.008)
	P2	13.98 ± 4.05	15.01 ± 3.78	15 ± 5.72	15.94 ± 6.28	.61(.002)	.10(.02)	.94(<.0001)
	P3	53.48 ± 15.72	55.3 ± 15.66	61.91 ± 8	60.05 ± 9.84	.19(.01)	.98(<.0001)	.15(.01)
	P4	22.95 ± 9.84	24.38 ± 11.94	18.27 ± 9.42	17.93 ± 9.86	.14(.01)	.79(.001)	.56(.002)
	P5	43.71 ± 11.31	45.27 ± 12.63	52.93 ± 12.1	54.94 ± 13.24	.05(.03)	.18(.01)	.86(.0002)

Bold font and * indicate significant values, α = .05.

**Table 3:** Descriptive statistics and fixed effects statistical results for sagittal plane joint range of motion (ROM) in the thorax-pelvis (degrees) for male and female professional and varsity football players during countermovement jump with arm swing (CMJ_AS_).

Males		Joint Angles Mean±SD%		*p*-value (ηp^2^)
Joint	Phase	Professional	Varsity	Category
Thorax-pelvis	P1	17.83 ± 6.32	14.19 ± 8.03	.13(.03)
	P2	30.92 ± 11.02	33.52 ± 9.97	.50(.007)
	P3	42.05 ± 8.56	44.75 ± 10.51	.47(.008)
	P4	12.07 ± 3.89	11.06 ± 4.06	.33(.01)
	P5	37 ± 13	28.12 ± 9.47	.06(.06)
**Females**		**Joint Angles Mean±SD%**		***p*-value (ηp**^**2**^)
**Joint**	Phase	**Professional**	**Varsity**	**Category**
Thorax-pelvis	P1	8.9 ± 6.36	9.38 ± 5.63	.82(.002)
	P2	23.56 ± 10.34	23.74 ± 12.73	.96(<.0001)
	P3	32.83 ± 6.12	32.51 ± 10.12	.92(.0001)
	P4	10.4 ± 5.94	8.72 ± 3.68	.26(.02)
	P5	26.32 ± 8.88	20.05 ± 6.03	.03(.10)*

Bold font and * indicate significant values, α = .05.

In males, post hoc comparisons revealed that varsity athletes exhibited greater knee and hip ROM during the P2 eccentric (*p* = .01; *p* = .004) and P3 concentric (*p* = .04; *p* = .001) phases, greater ankle ROM during the P3 concentric (*p* = .03) and P5 landing (*p* = .02) phases, lower knee ROM during the P4 flight phase (*p* = .03), and lower hip ROM during the P1 unloading phase (*p* < .0001) compared to professional athletes. In addition, a significant main effect of side was found for the knee during the P4 flight phase, which showed left-side dominance.

In females, significant differences were found only at the ankle during the P1 unloading phase with a medium effect size, with greater ROM in varsity athletes compared to professionals (*p* = .02). In addition, a significant difference was observed at the thorax-pelvis angle during the P5 landing phase with a large effect size, where professional female athletes exhibited greater ROM than varsity athletes (*p* = .03) (Table 3). No significant main effects of side were detected in females.

### Joint range of motion: AJ

Descriptive statistics (means and standard deviations) and mixed model fixed effects results for joint ROM during the AJ are presented in Tables 4 and 5 for both male and female athletes.

**Table 4:** Descriptive statistics and fixed effects statistical results for sagittal plane range of motion (ROM) in the knee, hip, and ankle (degrees) for male and female professional and varsity football players during approach jump (AJ).

		Joint Angles Mean±SD%				Fixed Effects		
Males		Professional		Varsity		*p*-value (ηp^2^)		
Joint	Phase	Left	Right	Left	Right	Category	Side	Category x Side
Knee	P2	61.5 ± 13.12	50.14 ± 12.18	62.94 ± 11.23	62.89 ± 19.02	.14(.02)	.004(.07)*	.01(.07)*
	P3	51.06 ± 23.41	38.69 ± 21.96	38.92 ± 22	38.72 ± 15.29	.37(.007)	.03(.04)*	.04(.03)*
	P4	71.29 ± 7.8	71.33 ± 9.32	70.72 ± 14.66	70.07 ± 15.47	.79(.001)	.82(.001)	.79(.001)
	P5	68.84 ± 22.59	69.38 ± 22.13	67.57 ± 14.35	66.08 ± 13.42	.76(.001)	.78(.001)	.56(.003)
Hip	P2	42.26 ± 8.8	35.24 ± 9.98	46.06 ± 8.54	46.54 ± 9.57	.01(.06)*	.02(.05)*	.01(.06)*
	P3	29.05 ± 19.28	39.91 ± 20.16	27.67 ± 17.79	27.97 ± 21.02	.32(.008)	.04(.04)*	.04(.03)*
	P4	47.01 ± 11.74	49.47 ± 7.97	49.81 ± 14.69	48.8 ± 15.97	.82(.0004)	.65(.002)	.24(.01)
	P5	46.43 ± 23.29	46.91 ± 24.25	44.97 ± 17.14	43.71 ± 16.58	.8(.001)	.77(.001)	.52(.003)
Ankle	P2	23.88 ± 7.64	20.24 ± 5.38	23.66 ± 4.81	20.66 ± 8.11	.98(.001)	.002(.08)*	.72(.001)
	P3	21.52 ± 11.88	18.12 ± 10.5	16.63 ± 7.74	18.5 ± 9.8	.5(.004)	.59(.003)	.06(.03)
	P4	50.86 ± 12.28	51.81 ± 9.07	52.61 ± 8.96	52.03 ± 10.78	.74(.001)	.93(.0001)	.65(.002)
	P5	56.1 ± 13.21	55.82 ± 12.25	61.93 ± 9.98	59.84 ± 11.98	.15(.002)	.53(.003)	.63(.02)
**Females**		**Professional**		**Varsity**		***p*-value (ηp**^**2**^)		
**Joint**	Phase	**Left**	**Right**	**Left**	**Right**	**Category**	**Side**	**Category x Side**
Knee	P2	66.53 ± 10.45	54.15 ± 9.43	54.6 ± 14.31	56.86 ± 15.26	.22(.01)	.01(.06)*	.0002(.11)*
	P3	52.53 ± 17.72	33.44 ± 19.31	41.66 ± 18.75	36.74 ± 16.91	.53(.003)	<.0001(.16)*	.01(.06)*
	P4	73.47 ± 10.03	75.3 ± 11.45	71.81 ± 10.9	68.49 ± 10.53	.23(.01)	.63(.002)	.1(.02)
	P5	78.97 ± 18.57	79.86 ± 21	68.38 ± 12.4	69.42 ± 12.44	.18(.02)	.59(.003)	.64(.002)
Hip	P2	48.18 ± 9.36	41.44 ± 8.27	41.98 ± 10.65	45.25 ± 7.72	.7(.001)	.15(.02)	<.0001(.13)*
	P3	25.23 ± 14.83	36.32 ± 26.37	33.26 ± 16.74	29.72 ± 17.88	.98(.001)	.26(.01)	**.01(.05)**
	P4	53.55 ± 9.88	55.26 ± 11.43	57.34 ± 11.49	56.46 ± 10.44	.56(.003)	.72(.001)	.28(.01)
	P5	65.89 ± 25.05	67.45 ± 25.01	49 ± 17.11	48.75 ± 15.27	.07(.03)	.49(.004)	.35(.008)
Ankle	P2	25.89 ± 4.36	20.18 ± 6.76	24.32 ± 4.48	23.39 ± 4.81	.62(.002)	<.0001(.14)*	.002(.08)*
	P3	17.73 ± 8.7	16.76 ± 6.66	12.85 ± 7.68	12.92 ± 6.4	.08(.03)	.66(.002)	.61(.002)
	P4	55.15 ± 9.26	57.37 ± 7.87	59.15 ± 9.72	57.16 ± 10.69	.53(.003)	.94(.0001)	.13(.02)
	P5	62.77 ± 6.05	64.53 ± 6.81	68.66 ± 6.93	68.84 ± 7.96	.06(.03)	.31(.009)	.28(.01)

Bold font and * indicate significant values, α = .05.

**Table 5:** Descriptive statistics and fixed effects statistical results for sagittal plane range of motion (ROM) in the thorax-pelvis (degrees) for male and female professional and varsity football players during approach jump (AJ).

Males		Joint Angles Mean±SD%		*p-*value (ηp^2^)
Joint	Phase	Professional	Varsity	Category
Thorax-pelvis	P2	24.3 ± 12.12	27.7 ± 6.86	0.40(.01)
	P3	23.21 ± 12.86	14.94 ± 9.38	0.06(.06)
	P4	40.62 ± 11.96	36.67 ± 11.9	0.36(.01)
	P5	38.12 ± 16.06	34.58 ± 8.64	0.51(.01)
**Females**		**Normalized EMG Amplitude Mean±SD%**		***p*-value (ηp**^**2**^)
**Joint**	Phase	**Professional**	**Varsity**	**Category**
Thorax-pelvis	P2	24.44 ± 9.11	17.88 ± 6.37	0.03(.08)*
	P3	14.52 ± 10.63	11.63 ± 8.08	0.48(.008)
	P4	35.57 ± 7.79	27.22 ± 7.15	0.001(.14)*
	P5	35.47 ± 10.61	23.15 ± 8.08	0.01(.13)*

Bold font and * indicate significant values, α = .05.

In males, a significant Category × Side interaction was found for both the knee and hip, with medium effect sizes (Table 4). Post hoc comparisons revealed greater knee ROM in the left limb compared to the right during the P2 eccentric (*p* = .001) and P3 concentric (*p* = .02) phases in professional athletes. A similar pattern was observed for the hip, with greater ROM in the left limb during the P2 eccentric phase (*p* = .002), but greater ROM in the right limb during the P3 concentric phase (*p* = .02) in professional athletes. Additionally, varsity athletes showed greater hip ROM during the P2 eccentric phase compared to professionals (*p* = .01). No significant differences were found for thorax-pelvis angle ROM in male athletes (Table 5).

In females, significant Category × Side interactions were found for the knee, hip, and ankle, all with medium to large effect sizes (Table 4). Post hoc comparisons indicated a significant asymmetry in knee ROM in professional athletes, with greater ROM in the left leg during both the P2 eccentric (*p* < .0001) and P3 concentric (*p* < .0001) phases. Professional female athletes also demonstrated greater hip and ankle ROM in the left leg compared to the right during the P2 eccentric phase (*p* = .001; *p* < .0001, respectively). Moreover, a significant main effect of category was found for the thorax-pelvis, with professional athletes exhibiting greater ROM compared to varsity athletes during the P2 eccentric, P4 flight, and P5 landing phases (Table 5).

### Mean muscle activation: CMJ_AS_

Descriptive statistics (means and standard deviations) and mixed model fixed effects results for mean muscle activation during the CMJ_AS_ are presented in Table 6 for both male and female athletes.

**Table 6:** Descriptive statistics and fixed effects statistical results for mean muscle activation (Normalized EMG amplitude %) for male and female professional and varsity football players during the countermovement jump with arm swing (CMJ_AS_).

		Normalized Mean EMG Amplitude Mean±SD%				Fixed Effects		
Males		Professional		Varsity		*p*-value (ηp^2^)		
Muscle	Phase	Left	Right	Left	Right	Category	Side	Category x Side
Biceps Femoris	P1	8.47 ± 5.8	10.75 ± 5.72	10.98 ± 7.62	9.85 ± 6.43	.81(.001)	.49(.004)	.06(.03)
	P2	47.65 ± 26.12	53.46 ± 27.94	46.08 ± 27.72	40.53 ± 29.94	.44(.004)	.90(.0001)	.02(.04)*
	P3	94.85 ± 37.08	102.43 ± 29.76	84.53 ± 42.58	86.88 ± 27.81	.25(.011)	.34(.008)	.37(.007)
	P4	53.88 ± 22.57	72.87 ± 34.62	50.55 ± 32.98	63.1 ± 37.68	.67(.002)	.004(.007)*	.32(.008)
	P5	35.68 ± 18.95	47.74 ± 22.47	30.88 ± 23.05	28.44 ± 15.68	.12(.02)	.06(.03)	.01(.06)*
Rectus Femoris	P1	43.26 ± 30.35	40.82 ± 27.6	37.62 ± 24.72	26.49 ± 24.52	.18(.02)	.12(.02)	.41(.006)
	P2	219.3 ± 130.97	191.98 ± 112.16	148.09 ± 89.75	128.18 ± 113.42	.16(.02)	.08(.03)	.9(.0001)
	P3	589.86 ± 243.58	458.75 ± 167.59	587.29 ± 217.25	564.34 ± 308.5	.63(.002)	.01(.06)*	.061(.03)
	P4	186.83 ± 55.35	163.72 ± 72.77	222.63 ± 95.48	168.99 ± 87.44	.53(.003)	.001(.10)*	.53(.003)
	P5	105.87 ± 53.26	80.65 ± 48.61	93.02 ± 60.71	72.85 ± 52.72	.55(.003)	.0003(.11)*	.52(.003)
Vastus Medialis	P1	40.64 ± 24.65	31.87 ± 23.99	36.67 ± 24.48	29.15 ± 21.27	.61(.002)	.03(.04)*	.88(.0002)
	P2	211.6 ± 128.19	209.89 ± 137.57	182.26 ± 116.87	217.26 ± 153.57	.71(.001)	.30(.009)	.02(.04)*
	P3	371.26 ± 162.67	345.07 ± 124.44	385.08 ± 134.48	401.23 ± 206.39	.62(.002)	.95(.00004)	.02(.05)*
	P4	166.4 ± 62.71	147.37 ± 60.97	190 ± 56.23	146.66 ± 82.42	.50(.004)	.002(.08)*	.31(.009)
	P5	100 ± 44.61	84.93 ± 42.06	97.24 ± 54.51	88.76 ± 55.52	.93(.001)	.05(.03)	.51(.004)
Gastrocnemius Medialis	P1	16.92 ± 9.56	15.51 ± 10.43	10.51 ± 7.2	10.38 ± 8.4	.21(.01)	.17(.02)	.95(.0001)
	P2	79.55 ± 40.39	78.2 ± 46.51	38.62 ± 22.87	45.09 ± 40.6	.01(.06)*	.79(.0006)	.65(.002)
	P3	212.31 ± 57.78	225.16 ± 71.18	215.87 ± 79.96	196.07 ± 50.46	.52(.003)	.79(.0006)	.10(.02)
	P4	124.1 ± 39.44	134.99 ± 44.74	144.62 ± 50.98	151.82 ± 53.04	.19(.02)	.29(.01)	.55(.003)
	P5	53.88 ± 23.14	56.87 ± 26.55	48.66 ± 29.54	64.27 ± 34.16	.66(.002)	.09(.02)	.14(.02)
**Females**		**Professional**		**Varsity**		***p*-value (ηp**^**2**^)		
**Muscle**	Phase	**Left**	**Right**	**Left**	**Right**	**Category**	**Side**	**Category x Side**
Biceps Femoris	P1	5.56 ± 2.83	7.02 ± 5.8	8.31 ± 5.59	10.44 ± 6.81	.10(.02)	.08(.03)	.98(<.001)
	P2	41.74 ± 26.72	38.42 ± 26.51	32.94 ± 20.43	48.43 ± 17.21	.87(.001)	.12(.02)	.02(.04)*
	P3	91.56 ± 44.94	82.5 ± 38.35	86.64 ± 32.52	112.33 ± 49.17	.39(.006)	.08(.03)	.002(.08)*
	P4	51.11 ± 32.34	64.11 ± 44.12	60.93 ± 34.69	67.74 ± 45.72	.57(.003)	.01(.06)*	.62(.002)
	P5	29.59 ± 15.58	35.59 ± 27.17	28.66 ± 18.14	42.9 ± 30.84	.57(.003)	.0004(.10)*	.16(.02)
Rectus Femoris	P1	46.6 ± 20.45	41.71 ± 24.81	43.56 ± 20.73	35.53 ± 25.37	.57(.003)	.06(.03)	.58(.003)
	P2	236.31 ± 101.63	227.77 ± 126.81	174.07 ± 97.5	221.77 ± 147.58	.51(.003)	.47(.03)	.33(.003)
	P3	488.09 ± 175.38	601.27 ± 256.73	465.98 ± 254.42	477.73 ± 214.4	.44(.005)	.01(.06)*	.09(.03)
	P4	204.95 ± 81.39	191.98 ± 97.31	183.56 ± 100.58	181.53 ± 93.72	.65(.002)	.54(.003)	.67(.002)
	P5	128.16 ± 46.15	121.34 ± 55.6	100.54 ± 69.02	93.28 ± 49.97	.27(.01)	.24(.01)	.67(.002)
Vastus Medialis	P1	32.18 ± 22.72	26.61 ± 20.78	31.75 ± 15.37	32.73 ± 21.82	.69(.001)	.52(.04)	.25(.01)
	P2	259.09 ± 119.73	258.39 ± 156.3	215.3 ± 93.68	238.68 ± 117.07	.43(.005)	.37(.007)	.55(.003)
	P3	493.46 ± 186.1	416.4 ± 206.24	447.83 ± 132.58	427.52 ± 167.77	.72(.001)	.01(.06)*	.11(.02)
	P4	205.98 ± 62.18	168.26 ± 88.36	175.92 ± 83.08	146.74 ± 68.82	.43(.005)	<.0001(.16)*	.67(.002)
	P5	155.22 ± 42.08	114.69 ± 55.53	95.96 ± 47.26	95.49 ± 54.75	.07(.03)	.002(.08)*	.003(.07)*
Gastrocnemius Medialis	P1	21.1 ± 11.59	14.68 ± 8.44	20.11 ± 12.09	19.06 ± 10.92	.68(.001)	.02(.05)*	.09(.03)
	P2	64.6 ± 34	65.4 ± 36.37	68.44 ± 50.54	62.27 ± 38.5	.78(.001)	.52(.003)	.48(.004)
	P3	206.8 ± 42.85	187.84 ± 56.93	214.73 ± 52.6	225 ± 59.57	.22(.01)	.57(.003)	.06(.03)
	P4	98.36 ± 24.96	99.72 ± 41.13	125.79 ± 45.35	137.28 ± 38.59	.02(.05)*	.16(.017)	.25(.01)
	P5	47.11 ± 21.85	52.51 ± 27.48	64.87 ± 32.66	62.32 ± 26.47	.17(.02)	.97(.00001)	.28(.01)

Bold font and * indicate significant values, α = .05.

In males, a significant Category × Side interaction was found for the biceps femoris and vastus medialis, both with medium effect sizes (Table 6). However, post hoc comparisons revealed only one significant asymmetry: greater biceps femoris activation in the right leg compared to the left during the P5 landing phase in professional athletes (*p* = .004). Additionally, a main effect of category was observed for the gastrocnemius medialis during the P2 eccentric phase, with higher mean activation in professional athletes compared to varsity athletes (*p* = .01). A main effect of side was also found across different phases, particularly for the rectus femoris, which showed greater activation in the left leg compared to the right.

In females, significant Category × Side interactions were found for the biceps femoris and vastus medialis, both showing medium effect sizes (Table 6). Post hoc comparisons indicated a significant asymmetry in biceps femoris activation in varsity athletes, with higher activation in the right leg during both the P2 eccentric (*p* = .04) and P3 concentric (*p* = .004) phases. Additionally, professional female athletes demonstrated greater vastus medialis activation in the left leg compared to the right during the P5 landing phase (*p* < .0001). Moreover, a main effect of category was observed for the gastrocnemius medialis during the P4 flight phase, with higher activation in varsity athletes compared to professional athletes (*p* = .02). A main effect of side was also found across various phases, particularly for the vastus medialis, which showed greater activation in the left leg compared to the right.

### Peak muscle activation: CMJ_AS_

Descriptive statistics (means and standard deviations) and mixed model fixed effects results for peak muscle activation during the CMJ_AS_ are presented in Table 7 for both male and female athletes.

**Table 7:** Descriptive statistics and fixed effects statistical results for peak muscle activation (Normalized EMG amplitude %) for male and female professional and varsity football players during the countermovement jump with arm swing (CMJ_AS_).

		Normalized peak EMG Amplitude Mean±SD%				Fixed Effects		
Males		Professional		Varsity		*p*-value (ηp^2^)		
Muscle	Phase	Left	Right	Left	Right	Category	Side	Category x Side
Biceps Femoris	P1	14.98 ± 10.4	21.91 ± 13.39	19.47 ± 14.22	14.68 ± 9.57	.61(.002)	.51(.004)	.001(.09)*
	P2	107.65 ± 42.01	100.91 ± 35.62	96.46 ± 41.31	83.11 ± 38.55	.26(.01)	.12(.02)	.26(.01)
	P3	131.95 ± 46.56	142.28 ± 38.57	124.37 ± 55.85	116.01 ± 45.04	.22(.01)	.73(.001)	.14(.02)
	P4	112.44 ± 51.9	135.66 ± 65.15	91.16 ± 63.3	115.07 ± 75.36	.39(.006)	.02(.05)*	.85(.0003)
	P5	108.72 ± 53.62	145.42 ± 98.42	84.49 ± 60.32	120.28 ± 88.68	.61(.002)	.002(.08)*	.31(.009)
Rectus Femoris	P1	79.95 ± 53.09	77.71 ± 44.12	55.64 ± 44.69	36.2 ± 31.51	.002(.08)*	.17(.02)	.33(.008)
	P2	475.65 ± 237.47	379.03 ± 203.35	375.74 ± 240.75	344.61 ± 277.66	.6(.002)	.04(.03)*	.49(.004)
	P3	788.38 ± 272.69	605.1 ± 191.22	819.31 ± 319.68	774.15 ± 416.88	.53(.003)	.0003(.12)*	.07(.03)
	P4	421.46 ± 126.32	367.42 ± 172.43	435.38 ± 220	371.95 ± 213.04	.98(<.001)	.004(.07)*	.96(<.0001)
	P5	400.79 ± 164.2	354.79 ± 190.26	327.07 ± 159.42	254.19 ± 166.4	.11(.02)	.01(.05)*	.94(.0001)
Vastus Medialis	P1	83.99 ± 58.66	93.6 ± 75.75	77.12 ± 66.71	53.24 ± 47.29	.40(.006)	.82(.0004)	.18(.02)
	P2	389.72 ± 238.68	363.22 ± 195.61	333.91 ± 180.45	443.73 ± 258.86	.97(<.001)	.11(.02)	<.0001(.1)*
	P3	537.15 ± 270.54	442.15 ± 160.2	499.47 ± 172.19	582.99 ± 301.88	.56(.003)	.77(.0007)	<.0001(.14)*
	P4	364.2 ± 156.23	306.17 ± 155.32	411.42 ± 207.8	344.24 ± 205.38	.50(.004)	.01(.06)*	.85(.0003)
	P5	356.92 ± 172.62	288.26 ± 166.15	344.16 ± 187.55	324.09 ± 208.5	.92(<.001)	.04(.03)*	.31(.009)
Gastrocnemius Medialis	P1	33.77 ± 23.29	28.12 ± 18.76	17.94 ± 17.68	16.57 ± 17.51	.1(.02)	.04(.04)*	.88(.0002)
	P2	201.84 ± 89.46	212.5 ± 107.08	137.5 ± 71.8	165.19 ± 116.39	.07(.03)	.45(.005)	.91(.0001)
	P3	297.56 ± 70.64	321.05 ± 108.87	277.46 ± 101.14	277.85 ± 84.08	.47(.004)	.6(.002)	.22(.01)
	P4	288.88 ± 114.61	302.99 ± 112.9	251.61 ± 105.23	235.72 ± 120.51	.32(.008)	.86(.0003)	.46(.005)
	P5	306.18 ± 117.51	314.97 ± 109.8	192.77 ± 81.21	296.97 ± 207.46	.27(.01)	.02(.04)*	.04(.04)*
**Females**		**Professional**		**Varsity**		***p-*value (ηp**^**2**^)		
**Muscle**	Phase	**Left**	**Right**	**Left**	**Right**	**Category**	**Side**	**Category x Side**
Biceps Femoris	P1	12.55 ± 7.69	13.69 ± 10.45	15.05 ± 11.26	17.95 ± 12.47	.36(.007)	.59(.003)	.95(.0003)
	P2	84.94 ± 50.33	69.94 ± 44.03	74.05 ± 42.81	93.89 ± 40.07	.70(.001)	.61(.002)	.22(.04)
	P3	113.41 ± 56.52	120.5 ± 64.82	115.12 ± 43.52	132.88 ± 57.5	.62(.002)	.04(.03)*	.25(.01)
	P4	87.16 ± 65.46	116.94 ± 88.43	99.03 ± 70.35	105.76 ± 83.59	.89(.0002)	.01(.05)*	.31(.009)
	P5	86.68 ± 69.25	90.34 ± 80.44	93.12 ± 74.82	125.6 ± 114.62	.48(.004)	.01(.06)*	.21(.01)
Rectus Femoris	P1	89.69 ± 39.7	69.83 ± 41.11	81.76 ± 48.95	58.21 ± 38.39	.5(.004)	.01(.06)*	.92(<.0001)
	P2	439.14 ± 206.38	481.59 ± 260.9	319.02 ± 185.81	382.87 ± 206.85	.19(.02)	.1(.02)	.94(<.0001)
	P3	602.86 ± 216.31	760.01 ± 359.84	614.27 ± 324.88	652.49 ± 328.44	.7(.001)	.01(.06)*	.1(.02)
	P4	377.91 ± 140.96	409.17 ± 215.89	353.67 ± 192.82	358.88 ± 167.6	.62(.002)	.5(.004)	.41(.006)
	P5	422.82 ± 191.82	444.2 ± 240.11	337.51 ± 228.9	378.34 ± 175.85	.37(.007)	.29(.01)	.85(.0003)
Vastus Medialis	P1	98.19 ± 62.56	83.63 ± 64.57	85.99 ± 58.37	101.19 ± 81.51	.88(.0002)	.93(.00006)	.13(.02)
	P2	458.44 ± 206.96	449.98 ± 251.58	391.35 ± 180.9	419.66 ± 183.27	.45(.005)	.52(.004)	.6(.002)
	P3	615.99 ± 256.71	523.69 ± 275.36	599.82 ± 220.16	572.81 ± 234.48	.93(<.0001)	.04(.04)*	.28(.01)
	P4	436.26 ± 154.22	324.91 ± 190.38	338.36 ± 175.23	275.81 ± 123.79	.29(.009)	<.0001(.14)*	.42(.006)
	P5	443.02 ± 158.15	343.87 ± 209.11	368.49 ± 184.54	339.84 ± 168.95	.54(.003)	.004(.07)*	.10(.02)
Gastrocnemius Medialis	P1	34.43 ± 18.6	26.53 ± 16.19	33.27 ± 21.36	29.14 ± 18.5	.81(.0005)	.02(.05)*	.58(.003)
	P2	151.58 ± 74.67	140.7 ± 61.71	160.06 ± 100.37	153.05 ± 83.01	.74(.0009)	.34(.008)	.84(.0004)
	P3	266.88 ± 69.1	235.46 ± 81.32	297.71 ± 68.54	309.37 ± 85.93	.03(.04)*	.42(.006)	.08(.03)
	P4	185.79 ± 63.31	221.67 ± 94.73	230.32 ± 96.92	270.4 ± 100.58	.09(.02)	.01(.05)*	.97(<.0001)
	P5	195.37 ± 92.31	223.04 ± 104.52	236.19 ± 115.35	279.77 ± 121.21	.16(.02)	.04(.04)*	.69(.001)

Bold font and * indicate significant values, α = .05.

In males, a significant Category × Side interaction was found for the biceps femoris, vastus medialis, and gastrocnemius medialis, with medium to large effect sizes (Table 7). Post hoc comparisons revealed a significant asymmetry in the biceps femoris, with greater peak activation in the right leg compared to the left during the P1 unloading phase in professional athletes (*p* = .02). An asymmetry was also observed in the vastus medialis, with greater activation in the left leg compared to the right during the P3 concentric phase (*p* = .01) in professional athletes. In varsity athletes, a significant asymmetry in vastus medialis peak activation was observed during the P2 eccentric (*p* = .002) and P3 concentric (*p* = .01) phases, with greater activation in the right leg. Similarly, an asymmetry in the gastrocnemius medialis was found during the P5 landing phase (*p* = .02), with higher peak activation in the right leg compared to the left, in varsity athletes. Additionally, a main effect of category was observed for the rectus femoris during the P1 unloading phase, with higher peak activation in professional athletes compared to varsity athletes (*p* = .002). A main effect of side was also found across various phases, particularly for the rectus femoris, which showed greater activation in the left leg compared to the right.

In females, no significant Category × Side interactions were observed. However, a significant main effect of category was found for the gastrocnemius medialis, with a small to medium effect size (Table 7). Post hoc comparisons indicated higher peak activation of the gastrocnemius medialis in varsity athletes compared to professional athletes during the P3 concentric phase (*p* = .03). Additionally, a significant main effect of side was found across various phases, particularly for the biceps femoris, which exhibited a right-side dominance, and vastus medialis, which showed a left-side dominance.

### Mean muscle activation: AJ

Descriptive statistics (means and standard deviations) and mixed model fixed effects results for mean muscle activation during the AJ are presented in Table 8 for both male and female athletes.

**Table 8:** Descriptive statistics and fixed effects statistical results for mean muscle activation (Normalized EMG amplitude %) for male and female professional and varsity football players during the approach jump (AJ).

		Normalized mean EMG Amplitude Mean±SD%				Fixed Effects		
Males		Professional		Varsity		*p*-value (ηp^2^)		
Muscle	Phase	Left	Right	Left	Right	Category	Side	Category x Side
Biceps Femoris	P2	12.71 ± 7.28	18.54 ± 9.19	13.47 ± 9.22	12.44 ± 9.18	.63(.002)	.23(.01)	.01(.07)*
	P3	67.27 ± 36.43	78.23 ± 31.53	89.68 ± 43.64	65.99 ± 38.11	.63(.002)	.36(.007)	.01(.06)*
	P4	90.1 ± 48.76	116.76 ± 50.36	102.06 ± 45.25	98.35 ± 50.9	.87(.0002)	.19(.01)	.08(.03)
	P5	37.23 ± 19.85	53.92 ± 23.12	37.9 ± 18.42	37.23 ± 24.64	.57(.003)	.01(.05)*	.002(.08)*
Rectus Femoris	P2	18.22 ± 10.5	16.4 ± 8.02	19.38 ± 8.91	20.11 ± 10.78	.36(.007)	.84(.0004)	.24(.01)
	P3	424.32 ± 216.03	339.92 ± 176.28	400.05 ± 281.54	370.88 ± 264.17	.96(.0001)	.04(.03)*	.34(.008)
	P4	555.59 ± 230.97	448.77 ± 211.75	605.17 ± 273.51	498.69 ± 244.51	.58(.003)	.0004(.1)*	.99(<.0001)
	P5	101.49 ± 40.46	72.95 ± 44.02	103.63 ± 39.85	76.58 ± 36.47	.68(.002)	<.0001(.23)*	.63(.002)
Vastus Medialis	P2	12.39 ± 7.06	8.02 ± 11.56	12 ± 9.01	14.78 ± 10.75	.67(.002)	.65(.002)	.05(.03)
	P3	320.52 ± 158.56	176.28 ± 265.54	328.54 ± 196.56	313.84 ± 246.58	.66(.002)	.06(.03)	.43(.005)
	P4	383.37 ± 165.33	211.75 ± 334.12	422.65 ± 180.54	386.1 ± 170.28	.42(.006)	.05(.03)	.63(.002)
	P5	93.09 ± 40.08	44.02 ± 72.87	98.66 ± 46.16	90.23 ± 43.21	.36(.007)	.004(.07)*	.12(.02)
Gastrocnemius Medialis	P2	20.41 ± 8.54	22.94 ± 8.39	29.28 ± 14.63	24.94 ± 9.95	.13(.02)	.83(.0004)	.05(.04)
	P3	164.92 ± 75.52	200.21 ± 127.31	181.32 ± 129.13	205.88 ± 117.3	.71(.001)	.09(.02)	.33(.008)
	P4	212.07 ± 79.73	230.99 ± 105.52	227.68 ± 101.3	241.85 ± 87.7	.92(<.0001)	.03(.04)*	.43(.005)
	P5	57.56 ± 17.42	67.83 ± 21.8	61.96 ± 25.33	61.12 ± 22.46	.87(<.0001)	.13(.04)	.12(.005)
**Females**		**Professional**		**Varsity**		***p*-value (ηp**^**2**^)		
**Muscle**	Phase	**Left**	**Right**	**Left**	**Right**	**Category**	**Side**	**Category x Side**
Biceps Femoris	P2	13.48 ± 7.75	17.33 ± 11.24	17.47 ± 9.31	16.68 ± 6.06	.52(.004)	.12(.02)	.12(.02)
	P3	68.57 ± 33.43	69.8 ± 46.46	74.73 ± 43.71	96 ± 47.31	.22(.01)	.05(.03)	.1(.02)
	P4	91.94 ± 32.94	114.75 ± 64.55	100.79 ± 45.64	115.13 ± 54.38	.64(.002)	.001(.09)*	.79(.006)
	P5	30.44 ± 17.23	38.88 ± 29.33	35.52 ± 24.9	35.84 ± 23.98	.66(.002)	.01(.06)*	.76(.0008)
Rectus Femoris	P2	17.57 ± 7.65	19.65 ± 10.89	24.92 ± 12.13	25.34 ± 12.57	.1(.02)	.17(.02)	.28(.01)
	P3	340.29 ± 150.82	449.75 ± 263.62	353.16 ± 223.99	373.5 ± 238.59	.75(.0009)	.01(.05)*	.04(.04)*
	P4	464.98 ± 172.64	556.47 ± 231.07	410.79 ± 230.13	445.7 ± 218.28	.29(.009)	.003(.07)*	.13(.02)
	P5	121.11 ± 44.41	123.19 ± 61.35	93.54 ± 53.14	94.44 ± 50.26	.12(.02)	.83(.0004)	.93(.0006)
Vastus Medialis	P2	14.66 ± 8.95	13.42 ± 8.61	18.14 ± 8.12	16.37 ± 10.54	.28(.01)	.53(.003)	.45(.005)
	P3	375.24 ± 191.35	326.24 ± 218.17	402.4 ± 179.18	392.05 ± 169.8	.89(.0002)	.32(.008)	.6(.002)
	P4	459.16 ± 158.54	419.65 ± 196.12	416.04 ± 140.61	389.39 ± 157.44	.41(.006)	.06(.03)	.58(.003)
	P5	122.97 ± 36.79	91.71 ± 36.76	91.16 ± 32.49	77.38 ± 29.9	.03(.04)*	.0001(.12)*	.14(.02)
Gastrocnemius Medialis	P2	31.3 ± 10.33	26.97 ± 12.63	30.61 ± 13.03	28.65 ± 5.73	.83(.0004)	.01(.05)*	.37(.007)
	P3	141.72 ± 54.4	127.8 ± 64.37	184.55 ± 116.44	171.33 ± 80.84	.1(.02)	.19(.02)	.95(.0001)
	P4	204.51 ± 60.55	192.16 ± 64.06	234.03 ± 81.01	243.63 ± 84.84	.08(.03)	.96(.0001)	.25(.01)
	P5	51.59 ± 19.17	51.38 ± 17.14	59.19 ± 29.83	63.1 ± 22.34	.13(.02)	.64(.002)	.6(.002)

Bold font and * indicate significant values, α = .05.

In males, a significant Category × Side interaction was found for the biceps femoris, with medium effect sizes (Table 8). Post hoc comparisons revealed significant asymmetry in biceps femoris mean activation, with greater activation in the right leg compared to the left during the P2 eccentric (*p* = .02) and P5 landing (*p* = .001) phases in professional athletes. In varsity athletes, greater activation was found in the left leg during the P3 concentric phase (*p* = .04). No significant main effect of category was observed for mean activation in males. However, a significant main effect of side was found across various phases, particularly for the rectus femoris, which showed greater activation in the left leg compared to the right.

In females, a significant Category × Side interaction was found only for the rectus femoris, showing a medium effect size (Table 8). Post hoc comparisons indicated a significant asymmetry in rectus femoris activation in professional athletes, with higher activation in the right leg during the P3 concentric phase (*p* = .01). Additionally, a main effect of category was observed for the vastus medialis, where professional female athletes demonstrated greater activation than varsity athletes during the P5 landing phase (*p* = .03). A main effect of side was also observed across multiple phases for all muscles. While the vastus medialis showed a left-side dominance, both the biceps femoris and rectus femoris demonstrated a right-side dominance.

### Peak muscle activation: AJ

Descriptive statistics (means and standard deviations) and mixed model fixed effects results for peak muscle activation during the AJ are presented in Table 9 for both male and female athletes.

**Table 9:** Descriptive statistics and fixed effects statistical results for peak muscle activation (Normalized EMG amplitude %) for male and female professional and varsity football players during the approach jump (AJ).

		Normalized peak EMG Amplitude Mean±SD%				Fixed Effects		
Males		Professional		Varsity		*p-*value (ηp^2^)		
Muscle	Phase	Left	Right	Left	Right	Category	Side	Category x Side
Biceps Femoris	P2	43.48 ± 29.99	58.05 ± 26.88	56.02 ± 39.32	58.99 ± 27.24	.48(.004)	.08(.03)	.2(.03)
	P3	111.81 ± 50.56	137.99 ± 64.89	130.72 ± 50.5	86.52 ± 34.26	.2(.01)	.37(.007)	0.001(.11)*
	P4	123.09 ± 59.39	160.47 ± 74.28	133.7 ± 63.75	129.96 ± 68.72	.62(.002)	.14(.02)	.07(.03)
	P5	145.05 ± 67.88	210.76 ± 106.8	143.87 ± 81.08	132.79 ± 78.88	.13(.02)	.03(.04)*	.003(.08)*
Rectus Femoris	P2	138.8 ± 104.86	147.07 ± 89.98	155.86 ± 114.96	140.77 ± 77.1	.77(.0007)	.94(.00006)	.42(.005)
	P3	727.39 ± 328.03	559.83 ± 261.29	640.01 ± 408.8	534.9 ± 331.56	.65(.002)	.002(.08)*	.46(.005)
	P4	742.36 ± 303.57	576.31 ± 271.38	789.97 ± 368.72	677.97 ± 367.29	.52(.003)	<.0001(.12)*	.61(.12)
	P5	508.29 ± 213.33	374.67 ± 196.25	491.54 ± 224.76	432.26 ± 260.4	.66(.002)	.0003(.11)*	.24(.01)
Vastus Medialis	P2	83.47 ± 55.89	61.36 ± 62.01	138.69 ± 121.82	114.94 ± 101.56	.07(.03)	.08(.03)	.9(.0001)
	P3	523.64 ± 216.14	450.69 ± 185.42	472.11 ± 241.71	435.78 ± 285.41	.76(.0008)	.03(.04)*	.64(.002)
	P4	504.76 ± 207.46	442.63 ± 181.03	541.53 ± 233.94	536.59 ± 267.04	.39(.006)	.21(.01)	.24(.01)
	P5	401.86 ± 152.84	324.02 ± 210.06	439.51 ± 205.91	420.41 ± 213.25	.29(.009)	.06(.03)	.25(.01)
Gastrocnemius Medialis	P2	124.84 ± 51.23	130.37 ± 65.59	125.02 ± 58.73	150.54 ± 74.15	.42(.006)	.31(.009)	.75(.0009)
	P3	278.77 ± 89.22	338.75 ± 145.84	285.62 ± 159.22	310.91 ± 120.36	.84(.0004)	.02(.05)*	.24(.01)
	P4	285.92 ± 97.62	304.99 ± 131.47	310.95 ± 129.85	325.57 ± 103.58	.76(.0008)	.11(.02)	.51(.004)
	P5	331.21 ± 146.61	396.69 ± 164.98	279 ± 160.63	303.12 ± 136.34	.27(.01)	.02(.04)*	.1(.02)
**Females**		**Professional**		**Varsity**		***p*-value (ηp**^**2**^)		
**Muscle**	Phase	**Left**	**Right**	**Left**	**Right**	**Category**	**Side**	**Category x Side**
Biceps Femoris	P2	54.58 ± 23.32	65.52 ± 35.71	67.1 ± 30.44	72.43 ± 29.38	.18(.02)	.09(.02)	.69(.001)
	P3	104.59 ± 39.62	105.25 ± 58.42	108.59 ± 52.64	134.06 ± 54.26	.32(.008)	.03(.04)*	.07(.03)
	P4	111.19 ± 36.83	136.98 ± 78.42	118.96 ± 49.82	127.99 ± 49.69	.84(.0003)	.002(.08)*	.87(.0002)
	P5	112.6 ± 96.4	147.99 ± 103.24	121.03 ± 84.74	150.08 ± 116.93	.47(.004)	.001(.09)*	.31(.009)
Rectus Femoris	P2	119.43 ± 77.72	160.6 ± 115.92	156.81 ± 127.17	214.53 ± 136.81	.25(.01)	.01(.07)*	.9(.0001)
	P3	529.76 ± 210.27	664.39 ± 309.36	539.24 ± 333.02	542.1 ± 268.23	.59(.002)	.02(.05)*	.02(.04)*
	P4	567.64 ± 213.94	733.39 ± 318.15	522.73 ± 296.93	535.99 ± 261.47	.26(.01)	.001(.08)*	.01(.06)*
	P5	519.73 ± 233.24	510.94 ± 282.98	389.8 ± 261.12	455.49 ± 270.32	.31(.008)	.23(.01)	.24(.01)
VastusMedialis	P2	98.81 ± 83.98	100.22 ± 84.62	114.74 ± 81.47	139.23 ± 94.94	.47(.004)	.16(.02)	.35(.007)
	P3	543.4 ± 198.65	516.52 ± 276.05	578.37 ± 198.13	573.81 ± 183.02	.59(.002)	.65(.002)	.77(.0007)
	P4	586.99 ± 214.58	530.87 ± 250.76	547.81 ± 181.58	506.4 ± 189.59	.7(.001)	.09(.02)	.7(.0009)
	P5	451.14 ± 166.08	397.94 ± 209.92	426.59 ± 198.54	361.14 ± 189.55	.5(.004)	.03(.04)	.98(<.0001)
Gastrocnemius Medialis	P2	169.2 ± 59.06	142.71 ± 52.64	141.26 ± 50.66	161.89 ± 66.81	.91(.0001)	.41(.006)	.01(.05)*
	P3	227.31 ± 68.9	208.15 ± 80.83	309.16 ± 144.17	264.54 ± 94.83	.02(.05)*	.05(.03)	.42(.006)
	P4	269.71 ± 68.14	249.8 ± 75.34	321.24 ± 103.88	308.65 ± 99.94	.05(.03)*	.21(.01)	.7(.001)
	P5	218.8 ± 102.34	255.14 ± 90.27	285.3 ± 159.35	321.68 ± 162.14	.07(.03)	.05(.03)	.78(.0006)

Bold font and * indicate significant values, α = .05.

In males, a significant Category × Side interaction was found for the biceps femoris, with medium to large effect sizes (Table 9). Post hoc comparisons revealed significant asymmetries in biceps femoris peak activation, with greater activation in the left leg compared to the right during the P3 concentric phase in varsity athletes (*p* = .01), and greater activation in the right leg compared to the left during the P5 landing phase in professional athletes (*p* = .001). No significant main effect of category was observed in males during this task. However, a significant main effect of side was found across various phases, particularly for the rectus femoris, which showed a left-side dominance, and the gastrocnemius medialis, which exhibited right-side dominance.

In females, significant Category × Side interactions were found for both the rectus femoris and gastrocnemius medialis, with medium effect sizes (Table 9). Post hoc comparisons revealed significant asymmetries in the rectus femoris, with greater peak activation in the right leg compared to the left during the P3 concentric (*p* = .01) and P4 flight (*p* = .0002) phases in professional athletes. For the gastrocnemius medialis, higher peak activation in the left leg compared to the right was observed during the P2 eccentric phase (*p* = .05) in professional athletes.

Moreover, a significant main effect of category was found for the gastrocnemius medialis, with a small to medium effect size. Post hoc comparisons indicated higher peak activation in varsity athletes compared to professional athletes during the P3 concentric (*p* = .02) and P4 flight (*p* = .05) phases. Additionally, a significant main effect of side was found across multiple phases, particularly for the biceps femoris and rectus femoris, both showing a right-side dominance.

### Performance and GRFz metrics: CMJ_AS_

Descriptive statistics (means and standard deviations) and mixed model fixed effects results for diverse performance and GRFz metrics during the CMJ_AS_ are presented in Table 10 for both male and female athletes.

**Table 10:** Descriptive statistics and fixed effects statistical results for performance and vertical ground reaction force (GRFz) metrics for male and female professional and varsity football players during countermovement jump with arm swing (CMJ_AS_).

Metric	Males		Category	Females		Category
	Professional	Varsity	p-value	Professional	Varsity	p-value
Jump Height (m)	0.41 ± 0.07	0.38 ± 0.05	.28(.02)	0.3 ± 0.05	0.23 ± 0.03	.0003(.21)*
Jump Momentum (kgm/s)	212.06 ± 17.55	181.35 ± 25.44	.003(.14)*	143.44 ± 22.28	127.05 ± 17.33	.08(.05)
RSI mod	0.11 ± 0.03	0.1 ± 0.02	.57(.005)	0.09 ± 0.01	0.07 ± 0.02	.01(.11)*
Jump Time (s)	3.84 ± 0.66	3.73 ± 0.7	.65(.003)	3.55 ± 0.49	3.41 ± 0.63	.4(.01)
Unloading time (s)	3.2 ± 0.66	2.8 ± 0.61	.06(.06)	2.94 ± 0.5	2.7 ± 0.64	.15(.03)
Yielding Time (s)	0.16 ± 0.05	0.25 ± 0.26	.05(.06)*	0.15 ± 0.04	0.18 ± 0.11	.3(.02)
Braking Time (s)	0.2 ± 0.06	0.35 ± 0.22	.03(.08)*	0.19 ± 0.04	0.21 ± 0.08	.25(.02)
Eccentric Time (s)	0.36 ± 0.09	0.6 ± 0.26	.001(.16)*	0.34 ± 0.06	0.4 ± 0.17	.24(.02)
Concentric Time (s)	0.28 ± 0.03	0.33 ± 0.04	.003(.14)*	0.28 ± 0.04	0.32 ± 0.07	.09(.05)
Landing Time (s)	0.36 ± 0.11	0.3 ± 0.13	.27(.02)	0.29 ± 0.1	0.27 ± 0.1	.83(.0008)
Unloading GRFz (%BW)	55.86 ± 14.54	31.8 ± 17.38	.001(.18)*	54.33 ± 12.26	38.44 ± 16.71	.01(.11)*
Unloading RFD (%BW/s)	−0.18 ± 0.06	−0.12 ± 0.07	**.03(.08)**	−0.19 ± 0.05	−0.15 ± 0.07	.07(.05)
Yielding RFD (BW/s)	4.03 ± 1.77	1.57 ± 1.61	.001(.18)*	3.91 ± 1.57	2.96 ± 2.17	.19(.03)
Braking GRFz (units of BW)	2.1 ± 0.16	1.74 ± 0.22	<.0001(.26)*	2.03 ± 0.21	1.85 ± 0.27	.06(.06)
Braking RFD (BW/s)	5.98 ± 1.93	2.99 ± 1.97	.0003(.2)*	5.93 ± 2.01	4.96 ± 2.83	.27(.02)
Concentric Avg GRFz (units of BW)	2.03 ± 0.11	1.86 ± 0.13	.002(.15)*	1.9 ± 0.12	1.71 ± 0.16	.003(.15)*
Momentum Landing (kg.m/s)	−226.23 ± 25.93	−190.58 ± 34.26	.01(.1)*	−148.04 ± 22.45	−131.96 ± 21.14	.12(.04)
Impact Peak GRFz (units of BW)	3.49 ± 0.52	3.89 ± 0.61	.11(.04)	4 ± 0.55	3.4 ± 0.46	.01(.12)*
Impact Avg GRFz (units of BW)	1.71 ± 0.28	2 ± 0.37	.04(.07)*	1.78 ± 0.31	1.66 ± 0.22	.29(.02)
Loading Rate (BW/s)	56.18 ± 21.41	58.99 ± 18.46	.9(.0003)	66.3 ± 22.85	43.3 ± 12.62	.01(.12)*

Bold font and * indicate significant values, α = .05. RFD = rate of force development, GRFz = vertical ground reaction force, BW = body weight.

In males, a significant category effect was observed across multiple force-related metrics, most with large effect sizes, indicating that professional athletes consistently outperformed their varsity counterparts. Professional males exhibited greater jump momentum, a larger percentage of body weight unloaded at the start of the jump, and applied more body weight–normalized force during both the braking and concentric phases. They also showed a higher rate of force development (RFD) during the unloading and eccentric (yielding and braking) phases compared to varsity males. Additionally, professionals demonstrated shorter durations in both the eccentric and concentric phases, reflecting a more efficient and explosive movement strategy. During the landing phase, they exhibited higher momentum at ground contact but experienced lower average vertical ground reaction forces (GRFz).

In females, fewer performance differences were observed between categories. However, professional athletes outperformed varsity athletes in several key metrics, including greater jump height, higher reactive strength index modified (RSI mod), and higher unloading and concentric GRFz. In contrast, varsity athletes exhibited lower peak impact forces and reduced loading rates during landing.

### Performance and GRFz metrics: Vertical AJ

Descriptive statistics (means and standard deviations) and mixed model fixed effects results for performance and GRFz metrics during the AJ are presented in Table 11 for both male and female athletes. Professional male athletes exhibited significantly greater peak force during the concentric phase and higher average impact GRFz during the landing phase compared to varsity athletes. In females, professionals demonstrated a higher jump height and greater peak braking force than their varsity counterparts.

**Table 11:** Descriptive statistics and fixed effects statistical results for performance and vertical ground reaction force (GRFz) metrics for male and female professional and varsity football players during approach jump (AJ).

Metric	Males		Category	Females		Category
	Professional	Varsity	*p*-value	Professional	Varsity	*p*-value
Jump Height (m)	0.48 ± 0.07	0.44 ± 0.05	0.19(.03)	0.371 ± 0.05	0.28 ± 0.03	<.0001(.32)*
Braking Peak GRFz (units of BW)	2.42 ± 0.86	1.94 ± 0.80	0.17(.03)	2.18 ± 0.48	1.59 ± 0.71	0.01(.10)*
Concentric Avg GRFz (units of BW)	1.65 ± 0.32	1.48 ± 0.27	0.11(.04)	1.44 ± 0.21	1.29 ± 0.21	0.07(.05)
Concentric Peak GRFz (units of BW)	2.93 ± 0.25	2.45 ± 0.46	0.004(.12)*	2.41 ± 0.34	2.21 ± 0.38	0.22(.02)
Impact Peak GRFz (units of BW)	2.85 ± 0.52	2.57 ± 0.61	0.16(.03)	2.51 ± 0.62	2.61 ± 0.55	0.58(.004)
Impact Avg GRFz (units of BW)	0.96 ± 0.11	0.84 ± 0.12	0.03(.07)*	0.84 ± 0.08	0.87 ± 0.09	0.55(.005)

Bold font and * indicate significant values, α = .05, GRFz = vertical ground reaction force, BW = body weight.

## Discussion

This study examined biomechanical, neuromuscular, and performance differences between Ecuadorian professional and varsity football players during the vertical AJ and CMJ_AS_. Among male athletes, professionals demonstrated a more explosive force production strategy, characterized by higher concentric and eccentric ground reaction forces, greater rates of force development, and shorter eccentric and concentric durations. These differences suggest distinct neuromuscular adaptations, even though both groups achieved similar jump heights and RSI mod values. Female professionals, by contrast, displayed superior jump performance, with significantly higher jump heights, RSI mod values, and thorax-pelvis ROM during landing, along with increased activation of the vastus medialis compared to varsity females. However, they also exhibited higher peak impact forces and loading rates. Varsity females showed higher gastrocnemius activation and lower force metrics than professional females, suggesting a compensatory strategy due to reduced proximal contribution. Neuromuscular and ROM asymmetries were more pronounced in professional athletes during the approach jump, particularly among females. These findings highlight the importance of evaluating multiple mechanical and neuromuscular variables, beyond jump height alone, to better understand injury risk profiles and inform targeted injury reduction strategies in football players. The following sections discuss these findings in greater depth.

### Males athletes: Professional vs varsity comparisons

Professional male athletes exhibited smaller knee and hip ROM during the eccentric and concentric phases of CMJ_AS,_ smaller hip ROM during the eccentric phase of AJ, and reduced ankle ROM during the concentric phase of CMJ_AS_ compared to varsity players. This finding aligns with previous research showing that excessive knee flexion (~90°) may reduce the efficiency of the stretch–shortening cycle (SSC) due to increased energy dissipation, whereas optimal knee flexion (~70°) enhances SSC utilization by improving elastic energy storage and subsequent power output [84,85]. The reduced ROM observed in professional male players may therefore reflect a movement strategy optimized for force efficiency, minimizing unnecessary joint excursion while maintaining high force output. This strategy may also be supported by the greater gastrocnemius activation observed during the eccentric phase of the CMJ_AS_ in professional males, suggesting enhanced control during deceleration. Additionally, the greater hip ROM observed during the unloading phase in professionals may indicate a more deliberate and controlled initiation of the countermovement, potentially facilitating improved eccentric control and more effective force generation, as supported by the increased rectus femoris peak activation observed in professionals, likely contributing to coordinated hip flexion and controlled descent.

These interpretations are further supported by the differences in force metrics observed across competitive levels. Professional males unloaded a higher percentage of BW at the initiation of movement and demonstrated significantly greater yielding RFD, braking GRFz, and braking RFD. A higher RFD during the braking phase reflects a more rapid deceleration of the center of mass, which can reduce elastic energy dissipation and improve energy transfer into the propulsive phase; factors that contribute positively to jump performance [86,87]. Furthermore, professional males exhibited higher concentric forces and shorter durations in both the eccentric and concentric phases of the CMJ_AS_, as well as in the concentric phase of the AJ, compared to varsity males. This suggests a greater capacity for rapid force production during propulsion, a key determinant of explosive performance and efficient energy utilization [88]. These force production and temporal characteristics suggest different propulsion strategies between competitive levels. Nevertheless, professional and varsity male players achieved similar jump heights in both the AJ and CMJ_AS_, likely due to the compensatory balance between higher force and shorter durations in professionals. This outcome aligns with previous research which found that jump height alone did not effectively distinguish between competitive levels in male athletes [89]. Taken together, these results reinforce the importance of evaluating detailed biomechanical and neuromuscular markers, rather than relying solely on jump height, as indicators of both explosive capacity and potential injury risk, particularly in male football players.

Regarding landing mechanics, increased knee and hip flexion at landing has been shown to reduce ACL injury risk by decreasing anterior tibial shear forces [90–92]. In the present study, no significant differences in knee or hip ROM were observed during the landing phase between professional and varsity athletes in either jump type. Reduced ROM during flight may limit effective pre-landing preparation, potentially compromising impact attenuation and movement control upon ground contact. Although increased ankle ROM has been associated with better landing mechanics and improved coordination strategies [93,94] it is important to note that the typical dorsiflexion range is approximately 40–50 degrees [95]. In the present study, the larger ankle ROM observed in varsity males may reflect joint hypermobility, which could contribute to ankle injuries [95]. This potential risk may be exacerbated by the higher average impact force and gastrocnemius peak activation asymmetry recorded in varsity males during CMJ_AS_ landing compared to professionals, suggesting reduced control over force absorption and a less stable landing strategy. Such profiles, particularly high ankle ROM, asymmetric muscle activation, and elevated impact forces, may predispose athletes to lateral ankle sprains or patellofemoral loading, especially in players lacking proximal strength control [96,97]. However, during the AJ the average impact force for varsity was lower than professionals with no differences in ROM during landing. This apparent discrepancy may reflect task-specific neuromuscular strategies. The CMJ_AS_ may elicit more forceful landings due to its controlled, stationary start, which allows athletes to optimize vertical force production. In contrast, the AJ involves a dynamic run-up, which may lead to altered landing mechanics, resulting in lower average impact forces despite similar ROM in varsity athletes. Overall, these findings highlight the importance of monitoring joint ROM and eccentric control during landing phases, as they may provide key indicators of neuromuscular readiness or fatigue. In particular, the reduced joint ROM observed in professionals may reflect a movement efficiency strategy but could also indicate increased joint stiffness and greater reliance on muscular stabilization. These adaptations, while potentially beneficial for performance, may elevate cumulative loading and fatigue-related injury risk [98], highlighting the need for individualized monitoring protocols.

### Female athletes: Professional vs varsity comparisons

In professional females, a larger percentage of body weight was unloaded at the initiation of the countermovement in the CMJ_AS._ Interestingly, this occurred despite varsity females exhibiting greater ankle ROM during the unloading phase. This suggests that professional athletes may rely more on proximal joint control (e.g., hip and trunk coordination) rather than increased distal joint mobility to generate force during the countermovement. Additionally, professional females exhibited substantially greater jump heights than varsity females in both the AJ and CMJ_AS_, a difference also reported by previous research [89] in female football players, though not observed in males, as discussed earlier. These performance differences may be partially explained by morphological and genetic predispositions, that are commonly accounted for in elite-level sport. Professional female athletes are often recruited through highly competitive pathways, where performance potential, which may include advantageous biomechanical or neuromuscular characteristics, plays a central role. In contrast, varsity-level athletes are typically younger, less experienced, and have had less access to elite training environments, which may contribute to their lower performance. Moreover, professional females achieved higher RSI mod values in the CMJ_AS_, indicating a better ability to rapidly develop force or explosive strength than their varsity counterparts [27]. In contrast, varsity females demonstrated greater gastrocnemius activation during the concentric and flight phases. This elevated distal activation may reflect a compensatory strategy for less efficient proximal force generation or less effective stretch-shortening cycle (SSC) utilization, as evidenced by their lower jump heights and force metrics.

Aside from this, fewer differences in ROM and force-related metrics were identified between professional and varsity females than among their male counterparts. However, notable distinctions emerged during the landing phase, where professional females demonstrated greater thorax-pelvis ROM in both the AJ and CMJ_AS_, possibly reflecting enhanced trunk control and a more integrated landing strategy. They also exhibited significantly greater peak impact forces and higher loading rates during landing, which may be partially attributed to their greater jump heights. However, these patterns could also suggest a stiffer landing strategy, likely relying more on muscular stabilization than joint excursion, which may elevate injury risk. This interpretation is further supported by the greater activation of the vastus medialis observed in professionals during both jump types. However, in the CMJ_AS_, this activation was asymmetric, which may increase injury risk due to uneven force distribution across limbs [99,100]. These neuromechanical characteristics are consistent with known injury mechanisms in elite female athletes, particularly those related to anterior knee pain and ACL injury [101,102]. Thus, they may suggest the need for individualized eccentric strength training or soft-landing drills to reduce injury risk. In addition, the high peak impact forces and observed asymmetries in professional females underscore the importance of trunk control and balanced bilateral strength development. Although asymmetries may arise from sport-specific demands, targeted bilateral training can help minimize maladaptive imbalances by ensuring both limbs develop similar strength and control capacities, potentially lowering ACL injury risk. This may be particularly important in elite female football, where neuromuscular control differences between limbs have been linked to increased injury susceptibility.

### Neuromuscular and kinematic asymmetries across sex and competitive level

Football inherently involves high levels of asymmetry due to the dominant kicking leg and repeated unilateral loading patterns during training and competition [103,104]. In this study, both professional and varsity male and female athletes exhibited muscle activation asymmetries, though their patterns varied across jump types and movement phases. These asymmetries were most frequently observed during the eccentric, concentric, and landing phases, and were primarily found in the biceps femoris and vastus medialis across groups. Notably, the most pronounced asymmetries were observed in male athletes and professional females, while varsity females showed biceps femoris asymmetry in only two CMJ_AS_ phases, with 16% and 26% side-to-side difference, respectively. Neuromuscular asymmetries have been previously documented in football players [105,106] and asymmetries exceeding 18% have been suggested to indicate significant imbalance [107] and increase the risk of injury [30,108]. In the present study, most asymmetries in professional males remained below the 18% threshold, except for peak activation of the vastus medialis during the CMJ_AS_ and the biceps femoris during the AJ. In contrast, all recorded asymmetries in varsity males and professional females exceeded 18%, with the largest differences (> 40%) observed in females in the mean activation of the vastus medialis in CMJ_AS_ and the mean and peak activation of the rectus femoris in the AJ. These patterns may reflect training-related neuromuscular adaptations, with the more pronounced asymmetries in varsity males and professional females potentially indicating a higher risk of injury due to imbalanced muscle activation and impaired interlimb coordination.

In terms of kinematic asymmetries, no ROM asymmetries were observed in CMJ_AS_. However, during the AJ, professional athletes exhibited more pronounced knee and hip ROM asymmetries during the eccentric and concentric phases. These differences may reflect task-specific adaptations to unilateral loading, but also suggest a higher neuromuscular demand and compromised movement stability during the AJ; factors that are associated with elevated injury risk if left unaddressed.

While some degree of asymmetries is inherent to football, excessive imbalances can impair force distribution and increase injury risk, underscoring the importance of targeted strength and neuromuscular training strategies [109,110]. Ultimately, while these insights enhance our understanding of movement efficiency and neuromuscular adaptations, they are especially valuable for identifying movement patterns that may predispose athletes to injury, supporting the integration of biomechanical screening into injury reduction programs.

### Limitations

The findings of this study should be interpreted in light of several limitations. First, while our sample size is limited, the use of repeated-measures mixed models and the detection of significant medium-to-large effects across several variables support the validity of the findings. Second, while the groups were demographically similar, other factors such as training intensity, exposure to structured conditioning programs, and competitive experience, may have influenced performance outcomes. Additionally, although testing was conducted at least two hours after a standardized morning training session, residual fatigue effects cannot be entirely ruled out and may have influenced performance measures. Consequently, the results may not be fully generalizable beyond the sample studied. In addition, as this research focused specifically on Ecuadorian football players, the applicability of these findings to athletes from other regions or training systems may be limited. Another limitation relates to the EMG normalization method: fixed submaximal loads may not fully account for individual strength differences, potentially affecting EMG comparability. Nevertheless, the study offers valuable insights into performance characteristics within this geographical and competitive context. Finally, this work should serve as a foundation for future research, particularly in the area of injury reduction strategies. Given the high incidence of muscle injuries in football and their impact on individual and team performance, continued investigation into biomechanical and neuromuscular risk factors is essential to inform more effective injury reduction strategies.

## Conclusion

This study provides comprehensive evidence of biomechanical, neuromuscular, and performance differences between Ecuadorian professional and varsity football players during CMJ_AS_ and AJ. Professional males demonstrated greater force production efficiency, including higher concentric and eccentric ground reaction forces, faster movement execution, and larger rates of force development, despite achieving similar jump heights to varsity athletes. Among females, professionals outperformed their varsity counterparts with greater jump heights, higher RSI mod values, and increased propulsive force output. However, they also exhibited higher peak impact forces, larger loading rates, and muscle activation asymmetries during landing; neuromechanical characteristics that have been associated with increased injury risk, particularly in female athletes. Across both sexes, joint ROM and muscle activation patterns varied by movement phase, with professionals generally relying more on proximal control and intermuscular coordination. Additionally, muscle activation asymmetries were more pronounced in varsity males and professional females, while ROM asymmetries were observed only in professional athletes, particularly during the approach jump, suggesting task-specific adaptations that may increase injury susceptibility if not properly addressed. These findings underscore the limitations of relying solely on performance metrics, such as jump height, to evaluate athletic ability, and highlight the value of assessing a broader set of biomechanical and neuromuscular variables. Ultimately, this study provides important neuromechanical benchmarks that can support the development of individualized training, screening, and injury reduction strategies. The insights gained are particularly relevant for practitioners working with elite South American football players and contribute to a growing body of evidence emphasizing the need for context-specific biomechanical profiling in high-performance sport.

## Supporting information

S1 File PLOS One's inclusivity in global research questionnaire. (DOCX)
